# The Associations of Vitamin D Status and Lifestyle Behaviors with General Obesity and Metabolically Unhealthy Obesity in Chinese Children and Adolescents

**DOI:** 10.3390/nu17040666

**Published:** 2025-02-13

**Authors:** Fangqu Liu, Yan Li, Chanhua Liang, Bingxuan Kong, Qian Zhang, Xingzhu Yin, Bangfu Wu, Jingfan Xiong, Ping Yao, Yuhan Tang, Yanyan Li

**Affiliations:** 1 Shenzhen Center for Chronic Disease Control, Shenzhen 518020, China; 2 Department of Nutrition and Food Hygiene, Hubei Key Laboratory of Food Nutrition and Safety, Ministry of Education Key Lab of Environment and Health, School of Public Health, Tongji Medical College, Huazhong University of Science and Technology, Wuhan 430030, China

**Keywords:** 25-hydroxyvitamin D, 24-Hour Movement Guidelines, children, obesity, metabolically unhealthy obesity

## Abstract

Background: Vitamin D and lifestyle behaviors are closely related to children’s health. However, current research on the combined influences of vitamin D and adherence to 24-Hour Movement Guidelines (24-HMG) on childhood obesity remains scarce. Our study aimed to examine individual and joint associations of vitamin D status and the number of recommendations for adhering to 24-HMG with obesity among Chinese children and adolescents. Methods: In this cross-sectional study, a total of 4625 participants from Shenzhen, China, were recruited. Vitamin D status was classified into two categories: adequacy and inadequacy. The 24-HMG was obtained from a validated questionnaire, including moderate-to-vigorous physical activity, screen time, and sleep duration. General obesity and metabolically unhealthy obesity (MUO) were the outcomes of this study. The logistic regression model was performed to examine the associations between vitamin D status, the number of guidelines met, and obesity. Results: Vitamin D inadequacy was associated with increased odds of general obesity and MUO, with adjusted odds ratios (95% CIs) of 1.551 (1.080–2.226) and 2.205 (1.319–3.686). Meeting 2–3 recommendations of 24-HMG was associated with decreased odds of general obesity, with adjusted odds ratios (95% CIs) of 0.777 (0.626–0.965). Compared with the vitamin D adequacy/meeting 2–3 guidelines group, the vitamin D inadequacy/meeting 0–1 guideline group was positively related to general obesity (adjusted odd ratio, 1.826 [95% CI: 1.167–2.857]) and MUO (2.160, 1.175–3.972). In addition, the population-attributable fractions of vitamin D inadequacy or/and meeting 0–1 guideline were 28.4% (95% CI: 2.5–54.4%) for general obesity and 42.3% (95% CI: 11.5–73.1%) for MUO. Conclusions: Our findings displayed that the combined associations of vitamin D inadequacy and meeting 0–1 recommendations of 24-HMG were linked to high odds of general obesity and MUO, respectively. Understanding these relationships could provide a theoretical basis for effective preventive strategies and interventions for childhood obesity.

## 1. Introduction

Childhood obesity has developed into a notable public health concern [1,2]. The implications of childhood obesity extend beyond physical health, profoundly affecting mental well-being [3,4]. Moreover, childhood obesity is highly correlated with the increased incidence of multiple chronic diseases during adulthood [5,6]. China has the largest number of children and adolescents with obesity globally, with the attendant health risks imposing a colossal strain on the healthcare system [7]. Of particular concern is the rising prevalence of obesity rates among Chinese children and adolescents, which is projected to reach 40% (girls) to 50% (boys) by 2035, according to the World Obesity Atlas 2023 [8]. Therefore, the identification of modifiable risk factors for obesity is crucial for the prevention and management of children with obesity.

Vitamin D exerts a significant impact on adipose tissue and adipocyte biology parameters through its modulation of adipogenesis, energy metabolism, and inflammation [9]. Findings from epidemiological studies have suggested an association between vitamin D deficiency and childhood obesity, even if the results were inconsistent [10,11,12]. Notably, previous studies have also shown that lifestyle behaviors were associated with children’s obesity [13,14,15,16]. However, researchers tend to focus on one or two behaviors. The 24-Hour Movement Guidelines (24-HMG) provide recommendations for three behaviors that occur during a single day, including moderate-to-vigorous physical activity (MVPA), screen time, and sleep duration [17,18]. Based on 24-HMG, a relatively comprehensive assessment of lifestyle can be performed. Current research findings were contradictory on the relationship between adherence to 24-HMG and obesity [19]. Adding to the complexity, vitamin D was closely related to lifestyle behaviors [20,21]. For example, physical activity has been reported to be associated with higher vitamin D levels, and sitting time was inversely correlated with vitamin D [22,23].

Given these conflicting findings and the potential interplay between vitamin D status and behaviors, it is imperative to investigate their joint association with obesity in Chinese children and adolescents. Nevertheless, not all individuals with obesity have an equally higher risk of diseases. Metabolically unhealthy obesity (MUO), a subset of obesity characterized by the presence of multiple metabolic abnormalities, is of particular concern as it confers a higher risk of cardiovascular morbidity and mortality [24]. To date, fewer studies explored the associations between vitamin D status and the number of recommendations for adhering to 24-HMG on general obesity among Chinese school-age children, and even fewer pertain to MUO in particular.

To fill these knowledge gaps, we aimed to assess the independent and joint relationships between vitamin D status and the number of guidelines met and general obesity and MUO among Chinese children and adolescents using representative sample data from the Evaluation and Monitoring on School-based Nutrition and Growth in Shenzhen (EMSNGS).

## 2. Methods

### 2.1. Study Design and Population

Participants in the study were selected from EMSNGS, an ongoing cohort carried out by the Shenzhen Center for Chronic Disease Control in Shenzhen, China. Additional details have been described previously [25]. This cross-sectional study was based on the project baseline data collected between October and December 2021. All participants signed written informed consent, and the research protocol was approved by the ethics committee of the Shenzhen Center for Chronic Disease Control (SZCCC-2021-037-01-PJ). In the present study, 4625 participants aged 6–17 years were included. Participants were excluded if they (1) failed to provide body mass index (BMI) and metabolic index; (2) failed to provide vitamin D level; (3) failed to provide 24-HMG components; (4) were aged ≥ 18 years; (5) had eaten breakfast on the survey day; or (6) had a history of chronic diseases, such as tumors and renal disease (Supplementary Figure S1).

### 2.2. Assessment of Vitamin D

Liquid chromatography-tandem mass spectrometry (LC-MS/MS) was considered the gold standard method for measuring serum levels of vitamin D [26]. In the present study, the concentration of 25-hydroxyvitamin D [25(OH)D] was also assessed by LC-MS/MS in a standardized laboratory, with a coefficient of variation (intra-assay and inter-assay) in below 10% (Agilent 6470, Agilent, Santa Clara, CA, USA). Serum 25(OH)D levels in this study were obtained by summing serum 25(OH)D_2_ and 25(OH)D_3_ levels. The status of 25(OH)D was classified as vitamin D inadequacy (<30 ng/mL) and vitamin D adequacy (≥30 ng/mL) according to the report released by the Endocrine Society and the Society for Adolescent Health and Medicine [27,28].

### 2.3. Assessment Components of 24-HMG

Details have been described previously [25]. In short, information on participants’ MVPA, sleep duration, and screen time was collected through a validated questionnaire by trained investigators. Meeting recommendations of MVPA was defined as engaging in MVPA at least 1 h per day. Adherence to screen time guidelines necessitates that daily screen time does not surpass 2 h. Furthermore, fulfilling sleep duration recommendations involves ensuring that participants aged 6 to 12 years sleep for 9 to 12 h per night, while those aged 13 to 18 years sleep for 8 to 10 h per night [29]. For each 24-HMG component, we assigned 1 point for a meet level and 0 points for a not meet level. Thus, the number of guidelines met was the sum of the points and ranged between 0 and 3, with higher scores indicating healthier lifestyles.

### 2.4. Assessment Covariates

Covariates were obtained through physical examinations or questionnaires, including demographic characteristics (e.g., age and sex), family status (e.g., maternal education, paternal education, parental obesity, parental history of metabolic disorders, and family income), pubertal status, tobacco exposure, and blood sampling season.

Parental obesity was defined as having a BMI of 28 kg/m^2^ or higher in either parent. Parental history of metabolic disorders was defined as the presence of hypertension, diabetes, or hyperlipidemia in either parent. Pubertal status categories were pre-puberty (Tanner Stage I), mid-puberty (Tanner Stages II and III), and post-puberty (Tanner Stages IV and V). Tobacco exposure encompasses both smokers and non-smokers, with the latter defined as individuals who inhale smoke exhaled by smokers for a cumulative duration of at least 15 min at least one day per week. The blood sampling season was divided into autumn and winter, from September to November and from December to February.

### 2.5. Outcome Ascertainment

The height and weight of each student were measured using a body weight measuring instrument (SH-200, Shanghe, Zhengzhou, China) that provides readings accurate to 0.1 cm for height and 0.1 kg for weight. BMI was calculated as weight divided by the square of height (kg/m^2^). Before the blood pressure measurements, each student was instructed to sit and relax for a period ranging from 15 to 30 min. Subsequently, their systolic blood pressure (SBP) and diastolic blood pressure (DBP) were recorded using an electronic sphygmomanometer (HBP-1320, Omron, Kyoto, Japan). Over 8 h of fasting blood samples were collected in the morning. These samples were then analyzed using an automated biochemical analyzer (AU5811, Beckman Coulter, Brea, CA, USA) to determine various biochemical parameters, including fasting blood glucose (FBG), high-density lipoprotein cholesterol (HDL-C), and triglyceride (TG). Comprehensive instrument calibration has been carried out, and stringent quality control procedures have been implemented.

General obesity was defined as having an age- and gender-specific BMI percentile ≥ 95th [30]. Based on the expert consensus and the population characteristics of Chinese children and adolescents, the definition of metabolic health included HDL-C > 1.03 mmol/L, TG < 1.7 mmol/L, SBP and DBP < P90, and FBG < 5.6 mmol/L. By combining obesity as diagnosed by BMI, participants can be divided into four categories: metabolically healthy non-obesity, metabolically unhealthy non-obesity, metabolically healthy obesity, and MUO. For the convenience of analysis, we combined the first three categories and referred to them as non-MUO [31,32]. MUO means children and adolescents with both obesity and metabolically unhealthy phenotypes.

### 2.6. Statistical Analysis

To determine the differences in basic characteristics by general obesity or MUO, statistical analysis was performed on the Wilcoxon rank sum test and the Chi-square test. In the logistic analyses, variables including age, sex, pubertal status, maternal education, paternal education, parental obesity, parental history of metabolic disorders, family income, tobacco exposure, and blood sampling season were adjusted as confounders. Regarding missing values of covariates, we used its mode to fill them. Imputation with the mode helped to retain the overall distribution and characteristics of the data. Multivariate adjusted odds ratios (ORs) and 95% confidence intervals (CIs) were calculated for general obesity or MUO. We used restricted cubic splines (RCSs) in logistics models to further explore the non-linear associations between 25(OH)D concentrations with general obesity and MUO. The *P*-trend was tested by treating the combination of vitamin D status and the number of guidelines met groups as a continuous variable. We assessed the interaction effect by including an interaction term between vitamin D status and the number of guidelines met in the logistic regression model adjusted for all other covariates. We also calculated the population-attributable fractions (PAFs) to estimate the proportion of general obesity or MUO that could theoretically be avoided if all participants who had vitamin D inadequacy or/and meeting 0 to 1 guideline were eliminated [33].

Stratified analyses were used to observe the relationship between the combination of vitamin D status and the number of guidelines met and general obesity or MUO in different subgroups (sex and age). In the sensitivity analyses, for missing data, we performed analyses that deleted participants with missing covariates (n = 347) or filled the missing variables by the multiple imputation method. We further adjusted for dietary vitamin D intake and the use of vitamin D supplements.

All data were analyzed by R software version 4.4.1 (R packages: rms, AF, mice). The difference was statistically significant with *p* < 0.05 in all analyses.

## 3. Results

### 3.1. Characteristics of Participants

A total of 4625 participants were included in the present analysis. Overall, the prevalence of general obesity and MUO were 9.34% and 6.40%, respectively. Students with obesity were more likely to be boys and had a higher proportion of parental obesity and parental metabolic abnormalities. In addition to the aforementioned characteristics, participants with MUO tended to be older (Table 1).

### 3.2. Associations Between Serum Vitamin D Status, General Obesity, and MUO

Table 2 shows the individual associations between vitamin D status, general obesity, and MUO using logistic regression models. In the fully adjusted model, the association of vitamin D inadequacy with general obesity was statistically significant (OR = 1.551, 95% CI: 1.080–2.226). Vitamin D inadequacy was associated with higher odds of MUO after adjusting for covariates (OR = 2.205, 95% CI: 1.319–3.686). We only discovered the non-linear relationship between 25(OH)D concentrations and MUO by RCS (Supplementary Figure S2). Moreover, multinomial logistic regression analysis was performed to evaluate the association of vitamin D inadequacy with metabolic phenotypes of obesity (Supplemental Table S1). Vitamin D inadequacy significantly increased the odds of being MUO rather than being MHNO by 2.112 times (95% CI: 1.251–3.564).

### 3.3. Associations Between the Number of Guidelines Met, General Obesity, and MUO

Compared with participants who met zero to one guideline, meeting two to three recommendations of 24-HMG was associated with decreased odds of general obesity (OR = 0.777, 95% CI: 0.626–0.965). There was no significant association between the number of guidelines met and MUO (Table 3). In the multinomial logistic regression model, meeting two to three recommendations of 24-HMG significantly decreased the odds of being MHO rather than being MHNO by 0.659 times (Supplemental Table S1).

### 3.4. Combined Associations of Serum Vitamin D Status and the Number of Guidelines Met with General Obesity and MUO

**Figure 1:**
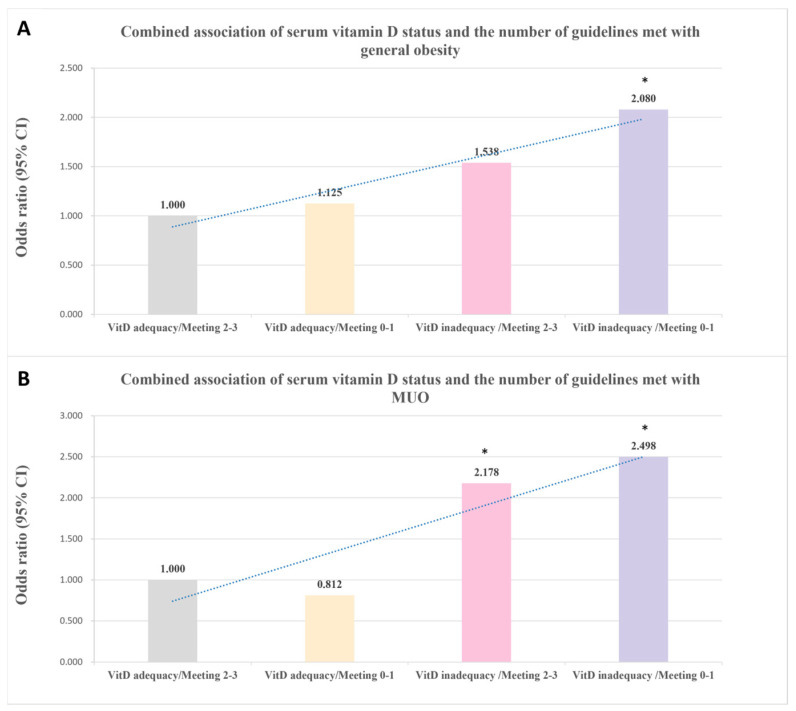
Combined associations of serum vitamin D status and the number of guidelines met with obesity. (**A**) General obesity; (**B**) MUO. The model adjusted for age, sex, pubertal status, maternal education, paternal education, parental obesity, parental history of metabolic disorders, family income, tobacco exposure, and blood sampling season. * *p*-value < 0.05. Abbreviations: CI, confidence interval; MUO, metabolic unhealthy obesity. VitD, vitamin D.

Figure 1 and Table 4 display the results of the logistic regression models performed for different combination groups. Individuals with vitamin D inadequacy and meeting zero to one guideline had significantly increased odds of general obesity compared with individuals with vitamin D adequacy and meeting two to three recommendations (OR = 1.826, 95% CI: 1.167–2.857, *P*-trend = 0.001). Additionally, compared with the reference group, participants with a combination of vitamin D inadequacy and meeting zero to one guideline were more likely to have a higher prevalence of MUO (OR = 2.160, 95% CI: 1.1.75–3.972, *P*-trend = 0.003). Subgroup analyses of the associations of serum vitamin D status and the number of guidelines met with general obesity and MUO are shown in Supplemental Table S2, and no multiplicative interaction between them (all *P* interactions > 0.05) was observed. In addition, the estimated PAFs of vitamin D inadequacy or/and meeting 0 to 1 guideline were 28.4% (95% CI: 2.5%, 54.4%) for general obesity and 42.3% (95% CI: 11.5%, 73.1%) for MUO (Table 4).

### 3.5. Subgroup Analysis and Sensitivity Analysis

Stratified analyses showed that the combined associations of vitamin D status and the number of guidelines met with general obesity and MUO were generally similar to the main result, particularly in boys and children (Supplemental Table S3). For missing data, we performed an analysis that deleted participants with missing covariates (Supplemental Table S4) and filled missing values by the multiple imputation method (Supplemental Table S5). We further adjusted for dietary vitamin D intake and vitamin D supplement use, and consistent results were observed (Supplemental Table S5).

## 4. Discussion

Using a large representative sample in Shenzhen, China, we systematically evaluated the individual and combined associations of vitamin D status and the number of recommendations for adhering to 24-HMG with general obesity and MUO. We found that participants with inadequate vitamin D had higher odds of being obese. Meeting two to three recommendations of 24-HMG was associated with decreased odds of general obesity. Joint analyses showed that compared with the vitamin D adequacy/meeting two to three guidelines group, the vitamin D inadequacy/meeting zero to one guideline group was associated with increased odds of general obesity and MUO. Our findings suggested that both adequate vitamin D levels and healthy behaviors may be potentially protective against childhood obesity, which has important public health implications.

Consistent with previous studies, our findings suggested that serum 25(OH)D status was associated with general obesity and MUO, respectively [34,35]. Some studies tended to report the negative associations of vitamin D with obesity and cardiometabolic risk factors in school-age children [10,36]. However, a cross-sectional study in Guangzhou, China, found that low vitamin D status was associated with general obesity in children and adolescents, independent of other cardiometabolic risk factors [37]. Considering these contradictory results, it may be due to differences in racial and diagnostic criteria. MUO is defined as obesity with unfavorable metabolic characteristics, and MUO can be used to observe the complex relationship between vitamin D and obesity from a novelty perspective. Vitamin D deficiency disturbs adipocytokine secretion, metabolism, lipid storage, adipogenesis, thermogenesis, the regulation of inflammation, and oxidative stress balance [38,39]. There is evidence that the underlying factors contributing to the development of metabolic disorders in childhood obesity may mainly be due to adipose tissue inflammation and adipogenesis dysfunction [40]. A prospective study showed that vitamin D might prevent the development of MUO [32]. More robust researches are needed on whether vitamin D can reverse MUO.

There is a growing awareness of the necessity to explore 24-HMG as a whole, given the interaction between lifestyle behaviors [41]. Children and adolescents who met all the guidelines of 24-HMG were more likely to have lower risks of being obese, but the results are contradictory. Consistent with our results, Huang et al. reported an inverse association between adherence to 24-HMG and the risk of being overweight and obese in children and adolescents after a mean follow-up of 4.7 years [42]. However, two studies showed that meeting the guidelines was not associated with obesity [43,44]. Differences in measurement tools to assess components of 24-HMG may be a potential reason. Furthermore, our results showed that children and adolescents who are obese with metabolic abnormalities may benefit from meeting two to three recommendations of 24-HMG, even if not statistically significant. To our knowledge, the present study is one of the few exploring the association between adherence to 24-HMG and MUO. In the future, to elucidate the relationship between meeting the 24-HMG and obesity in children and adolescents, it would be valuable to conduct prospective cohort studies or randomized controlled trials utilizing more accurate assessment methods for body fat or 24-HMG.

Inadequate 25(OH)D levels and unhealthy lifestyle behaviors often co-exist in children and adolescents, and they are both risk factors for obesity. Previous studies have focused on the joint association between physical activity and vitamin D with healthy outcomes in school-age children [45]. The underlying mechanism may be related to the synthesis pathway of vitamin D; serum 25(OH)D concentrations can be increased by sunlight exposure during physical activity [20]. It is important to note that not only can lifestyle behaviors influence each other, but behaviors are also related to the concentration of vitamin D in the body. For example, daytime physical activity was associated with lower total sleep time [46]. Excessive screen time was inversely associated with vitamin D levels, possibly due to decreased physical activity [47]. Vitamin D deficiency was associated with sleep disorders, and the mechanism is mainly involved in serotonergic and dopaminergic pathways [48]. Our findings revealed significant effects of the combinations of the number of guidelines met and vitamin D status for obesity. In subgroup analyses, we found that the association was robust in children and boys. Different age- and sex-specific in vivo hormonal and behavioral patterns may account for this difference.

Today’s patients with obesity are often diagnosed and treated in a very similar way, as if they all suffer from the same degree of disease [49]. Indeed, individuals with obesity can exhibit different metabolic phenotypes [50]. MUO can define high-risk individuals with obesity through the assessment of metabolic status, thereby facilitating the graded management of children with obesity. Our study results highlighted the importance of adequate vitamin D levels and healthy lifestyle behaviors in children and adolescents who are obese with concomitant metabolic abnormalities. In addition, we hypothesized that meeting recommendations of 24-HMG might alter vitamin D levels to reduce obesity, but the result of the mediation analysis was not statistically significant (Supplementary Figure S3). Therefore, we believe that further researches (e.g., prospective cohort studies or randomized controlled trials) are required to improve our understanding of how 24-HMG and vitamin D affect obesity in children and youths at the same time.

The major strength of this study is that the joint association between serum vitamin D status and adherence to 24-HMG with general obesity and MUO was explored for the first time. Nevertheless, we also acknowledge several limitations. Firstly, this study is a cross-sectional study and cannot clarify the causal relationship between 25(OH)D status and lifestyle behaviors and obesity. Secondly, information on 24-HMG was self-reported; thus, measurement errors were inevitable. Thirdly, because primary school students in grades one to three were surveyed by a simplified dietary questionnaire, no energy adjustment was made in the analysis. Finally, the population in this study came from Shenzhen, which is located in the developed coastal area of China and has a low latitude. Therefore, our sample can represent the children and adolescents in the developed coastal areas of China, and the representative of Chinese children and adolescents in the less developed areas of high latitude was poor. For the above reasons, the results should be cautiously interpreted.

## 5. Conclusions

Our findings observed that the combined associations of inadequate vitamin D levels and meeting zero or one recommendation of 24-HMG were associated with increased odds of general obesity and MUO. These results suggested that lifestyle behaviors and vitamin D status may play a role in childhood obesity. We recommended that while vitamin D levels are being measured, schools and families should monitor and motivate children and adolescents to meet as many of the 24-HMG recommendations as possible. Moreover, further research is needed to confirm causation and inform targeted interventions.

## Figures and Tables

**Table 1:** Basic characteristics grouped by general obesity or MUO status among 4625 individuals.

Variables	Non-General Obesity (N = 4193)	General Obesity (N = 432)	*p*-Value	Non-MUO (N = 4329)	MUO (N = 296)	*p*-Value
Age (years)	11.94 [9.13, 14.73]	11.54 [9.21, 14.12]	0.222	11.83 [9.07, 14.68]	12.79 [10.13, 15.07]	<0.001
Sex			<0.001			<0.001
Boys	2251 (53.68)	294 (68.06)		2322 (53.64)	223 (75.34)	
Girls	1942 (46.32)	138 (31.94)		2007 (46.36)	73 (24.66)	
Puberty status			0.101			<0.001
Pre-puberty	1334 (31.81)	127 (29.40)		1397 (32.27)	64 (21.62)	
Mid-puberty	910 (21.70)	113 (26.16)		954 (22.04)	69 (23.31)	
Post-puberty	1949 (46.48)	192 (44.44)		1978 (45.69)	163 (55.07)	
Maternal education			0.704			0.893
Junior college or below	3127 (74.58)	318 (73.61)		3226 (74.52)	219 (73.99)	
College or above	1066 (25.42)	114 (26.39)		1103 (25.48)	77 (26.01)	
Paternal education			0.063			0.32
Junior college or below	2868 (68.40)	276 (63.89)		2951 (68.17)	193 (65.20)	
College or above	1325 (31.60)	156 (36.11)		1378 (31.83)	103 (34.80)	
Parental obesity			<0.001			<0.001
Yes	416 (9.92)	95 (21.99)		449 (10.37)	62 (20.95)	
No	3777 (90.08)	337 (78.01)		3880 (89.63)	234 (79.05)	
Parental history of metabolic disorders			0.001			0.011
Yes	519 (12.38)	79 (18.29)		545 (12.59)	53 (17.91)	
No	3674 (87.62)	353 (81.71)		3784 (87.41)	243 (82.09)	
Family income			0.087			0.123
<50,000 CNY	297 (7.08)	32 (7.41)		306 (7.07)	23 (7.77)	
~250,000 CNY	2287 (54.54)	212 (49.07)		2356 (54.42)	143 (48.31)	
≥250,000 CNY	1609 (38.37)	188 (43.52)		1667 (38.51)	130 (43.92)	
Tobacco exposure			0.477			0.128
Yes	611 (14.57)	69 (15.97)		627 (14.48)	53 (17.91)	
No	3582 (85.43)	363 (84.03)		3702 (85.52)	243 (82.09)	
Blood sampling season			0.031			0.021
Autumn	2910 (69.40)	322 (74.54)		3007 (69.46)	225 (76.01)	
Winter	1283 (30.60)	110 (25.46)		1322 (30.54)	71 (23.99)	
Vitamin D (ng/mL)	21.39 [17.30, 26.01]	21.73 [17.98, 26.06]	0.378	21.45 [17.36, 26.10]	21.12 [17.41, 24.80]	0.186
Number of guidelines met			0.198			0.099
Meeting 0–1	1743 (41.57)	194 (44.91)		1799 (41.56)	138 (46.62)	
Meeting 2–3	2450 (58.43)	238 (55.09)		2530 (58.44)	158 (53.38)	

Quantitative variables were shown as the median [interquartile range]. Categorical variables were presented as numbers (percentages). Abbreviations: MUO, metabolic unhealthy obesity.

**Table 2:** Associations between serum vitamin D status, general obesity, and MUO.

	Crude	Model 1	Model 2
	OR (95% CI)	OR (95% CI)	OR (95% CI)
General obesity			
Vitamin D			
Adequacy	1.000 (reference)	1.000 (reference)	1.000 (reference)
Inadequacy	1.334 (0.944, 1.886)	1.541 (1.080, 2.199)	1.551 (1.080, 2.226)
*p*-value	0.103	0.017	0.017
MUO			
Vitamin D			
Adequacy	1.000 (reference)	1.000 (reference)	1.000 (reference)
Inadequacy	2.138 (1.299, 3.520)	2.134 (1.283, 3.549)	2.205 (1.319, 3.686)
*p*-value	0.003	0.003	0.003

Model 1 adjusted for age and sex. Model 2 was further adjusted for pubertal status, maternal education, paternal education, parental obesity, parental history of metabolic disorders, family income, tobacco exposure, and blood sampling season. Abbreviations: CI, confidence interval; OR, odds ratio.

**Table 3:** Associations between the number of guidelines met, general obesity, and MUO.

	Crude	Model 1	Model 2
	OR (95% CI)	OR (95% CI)	OR (95% CI)
General obesity			
Number of guidelines met			
Meeting 0–1	1.000 (reference)	1.000 (reference)	1.000 (reference)
Meeting 2–3	0.873 (0.715, 1.065)	0.772 (0.623, 0.957)	0.777 (0.626, 0.965)
*p*-value	0.181	0.018	0.023
MUO			
Number of guidelines met			
Meeting 0–1	1.000 (reference)	1.000 (reference)	1.000 (reference)
Meeting 2–3	0.814 (0.643, 1.031)	0.873 (0.675, 1.128)	0.876 (0.677, 1.134)
*p*-value	0.088	0.299	0.316

Model 1 adjusted for age and sex. Model 2 was further adjusted for pubertal status, maternal education, paternal education, parental obesity, parental history of metabolic disorders, family income, tobacco exposure, and blood sampling season. Abbreviations: CI, confidence interval; OR, odds ratio; MUO, metabolic unhealthy obesity.

**Table 4:** Combined associations of serum vitamin D status and the number of guidelines met with obesity.

	Crude	Model 1	Model 2	PAF ^a^
	OR (95% CI)	OR (95% CI)	OR (95% CI)	(%)
General obesity				
VitD adequacy/Meeting 2–3	1.000 (reference)	1.000 (reference)	1.000 (reference)	**28.4 (2.5, 54.4)**
VitD adequacy/Meeting 0–1	0.954 (0.461, 1.976)	0.995 (0.479, 2.065)	0.925 (0.442, 1.933)	
VitD inadequacy/Meeting 2–3	1.238 (0.816, 1.878)	1.410 (0.925, 2.150)	1.377 (0.896, 2.115)	
VitD inadequacy/Meeting 0–1	1.419 (0.932, 2.162)	**1.879 (1.206, 2.927)**	**1.826 (1.167, 2.857)**	
*P*-trend	0.043	0.001	0.001	
MUO				
VitD adequacy/Meeting 2–3	1.000 (reference)	1.000 (reference)	1.000 (reference)	**42.3 (11.5, 73.1)**
VitD adequacy/Meeting 0–1	0.716 (0.230, 2.231)	0.693 (0.222, 2.168)	0.643 (0.205, 2.019)	
VitD inadequacy/Meeting 2–3	**1.785 (1.001, 3.183)**	**1.832 (1.021, 3.286)**	**1.840 (1.019, 3.325)**	
VitD inadequacy/Meeting 0–1	**2.182 (1.220, 3.900)**	**2.162 (1.179, 3.966)**	**2.160 (1.175, 3.972)**	
*P*-trend	0.001	0.003	0.003	

Model 1 adjusted for age and sex. Model 2 was further adjusted for pubertal status, maternal education, paternal education, parental obesity, parental history of metabolic disorders, family income, tobacco exposure, and blood sampling season. ^a^ PAF was calculated based on Model 2. The bolded result means that the *p*-value is less than 0.05. Abbreviations: CI, confidence interval; OR, odds ratio; MUO, metabolic unhealthy obesity; VitD, vitamin D; PAF, population-attributable fraction.

## Data Availability

The authors do not have permission to share data due to confidentiality and privacy considerations.

## Supplementary Materials

The following supporting information can be downloaded at https://www.mdpi.com/article/10.3390/nu17040666/s1: Table S1. Associations between serum vitamin D status, the number of guidelines met, and metabolic phenotypes of obesity by multinomial logistic regression. Table S2. Subgroup analyses of the associations of serum vitamin D status and the number of guidelines met with general obesity and MUO. Table S3. Stratified analyses of combined associations of serum vitamin D status and the number of guidelines met with general obesity and MUO. Table S4. Combined associations of serum vitamin D status and the number of guidelines met with general obesity and MUO after excluding the individuals that missing covariates values (n = 4278). Table S5. Sensitivity analyses of combined associations of serum vitamin D status and the number of guidelines met with general obesity and MUO. Figure S1. Flowchart for the participants’ selection. Abbreviations: EMSNGS, Evaluation and Monitoring on School-based Nutrition and Growth in Shenzhen; FBG, fasting blood glucose; HDL-C, high-density lipoprotein cholesterol; MVPA, moderate-to-vigorous physical activity; ST, screen time; SLP, sleep duration; TG, triglyceride. Figure S2. Nonlinear dose-response relationship between vitamin D concentrations and general obesity or MUO in children and adolescents. (A) General obesity; (B) MUO. Model adjusted for age, sex, pubertal status, maternal education, paternal education, parental obesity, parental history of metabolic disorders, family income, tobacco exposure, and blood sampling season. Abbreviations: CI, confidence interval; MUO, metabolic unhealthy obesity. Figure S3. Parallel mediation analyses of the association between adherence to 24-HMG and general obesity or MUO mediated by vitamin D concentrations. (A) General obesity; (B) MUO. Model adjusted for age, sex, pubertal status, maternal education, paternal education, parental obesity, parental history of metabolic disorders, family income, tobacco exposure, and blood sampling season. Abbreviations: 24-HMG, 24-Hour Movement Guidelines; CI, confidence interval; DE, direct effect; IE, indirect effect; MUO, metabolic unhealthy obesity; Proportion of mediation  =  IE/DE + IE.
