# Supplementary material for: The Associations of Vitamin D Status and Lifestyle Behaviors with General Obesity and Metabolically Unhealthy Obesity in Chinese Children and Adolescents
Source: Nutrients. 2025 Feb 13;17(4):666. doi: 10.3390/nu17040666 (PMC11858261; doi:10.3390/nu17040666)
Supplement: Supplementary file 1 [file nutrients-17-00666-s001.zip › nutrients-3454894-supplementary.pdf]

The Associations of Vitamin D Status and Lifestyle Behaviors with General Obesity and Metabolically Unhealthy Obesity in Chinese Children and Adolescents

Supplementary material

Tables:

Supplementary Table 1. Associations between serum vitamin D status, the number of guidelines met, and metabolic phenotypes of obesity by multinomial logistic regression.

Supplementary Table 2. Subgroup analyses of the associations of serum vitamin D status and the number of guidelines met with general obesity and MUO.

Supplementary Table 3. Stratified analyses of combined associations of serum vitamin D status and the number of guidelines met with general obesity and MUO.

Supplementary Table 4. Combined associations of serum vitamin D status and the number of guidelines met with general obesity and MUO after excluding the individuals that missing covariates values (n=4278).

Supplementary Table 5. Sensitivity analyses of combined associations of serum vitamin D status and the number of guidelines met with general obesity and MUO.

Figures:

Supplementary Figure 1. Flowchart for the participants' selection.

Supplementary Figure 2. Nonlinear dose-response relationship between vitamin D concentrations and general obesity or MUO in children and adolescents.

Supplementary Figure 3. Parallel mediation analyses of the association between adherence to 24-HMG and general obesity or MUO mediated by vitamin D concentrations.

Supplementary Table S1. Associations between serum vitamin D status, the number of guidelines met, and metabolic phenotypes of obesity by multinomial logistic regression.

	MHNO<br>OR (95%CI)	MUNO<br>OR (95%CI)	MHO<br>OR (95%CI)	MUO<br>OR (95%CI)
Vitamin D inadequacy				
Model 1	1.000 (reference)	0.930 (0.751, 1.151)	1.035 (0.632, 1.696)	**2.072 (1.235, 3.475)**
Model 2	1.000 (reference)	0.907 (0.729, 1.129)	0.986 (0.595, 1.633)	**2.112 (1.251, 3.564)**
Meeting 2-3 guidelines				
Model 1	1.000 (reference)	1.125 (0.975, 1.298)	**0.661 (0.458, 0.954)**	0.905 (0.694, 1.180)
Model 2	1.000 (reference)	1.101 (0.952, 1.273)	**0.659 (0.455, 0.955)**	0.903 (0.691, 1.181)

Model 1 adjusted for age, sex;

Model 2 was further adjusted for pubertal status, maternal education, paternal education, parental obesity, parental history of metabolic disorders, family income, tobacco exposure, and blood sampling season.

Abbreviations: CI, confidence interval; OR, odds ratio, MHNO, metabolically healthy non-obesity; MUNO, metabolically unhealthy non-obesity; MHO, metabolically healthy obesity; MUO, metabolically unhealthy obesity.

Supplementary Table S2. Subgroup analyses of the associations of serum vitamin D status and the number of guidelines met with general obesity and MUO.

Group		General obesity<br>OR (95%CI)	MUO<br>OR (95%CI)
Vitamin D adequacy (n= 516)	Meeting 0-1	1.000 (reference)	1.000 (reference)
	Meeting 2-3	1.145 (0.537, 2.443)	1.566 (0.473, 5.193)
Vitamin D inadequacy (n= 4109)	Meeting 0-1	1.000 (reference)	1.000 (reference)
	Meeting 2-3	**0.753 (0.599, 0.947)**	0.845 (0.647, 1.105)
Meeting 0-1 (n= 1937)	Vitamin D adequacy	1.000 (reference)	1.000 (reference)
	Vitamin D inadequacy	**2.400 (1.228, 4.689)**	**4.179 (1.483, 11.778)**
Meeting 2-3 (n= 2688)	Vitamin D adequacy	1.000 (reference)	1.000 (reference)
	Vitamin D inadequacy	1.278 (0.829, 1.971)	1.680 (0.924, 3.054)
P-interaction		0.358	0.314

Model adjusted for age, sex, pubertal status, maternal education, paternal education, parental obesity, parental history of metabolic disorders, family income, tobacco exposure, and blood sampling season.

A cross-product interaction term was included in the logistic regression model to assess multiplicative interaction.

Abbreviations: CI, confidence interval; OR, odds ratio; MUO, metabolic unhealthy obesity.

Supplementary Table S3. Stratified analyses of combined associations of serum vitamin D status and the number of guidelines met with general obesity and MUO.

Subgroup	General obesity<br>OR (95%CI)	MUO<br>OR (95%CI)
Sex		
Boys		
VitD adequacy/Meeting 2-3	1.000 (reference)	1.000 (reference)
VitD adequacy/Meeting 0-1	0.905 (0.363, 2.256)	0.650 (0.174, 2.427)
VitD inadequacy/Meeting 2-3	1.647 (0.977, 2.778)	**2.070 (1.055, 4.060)**
VitD inadequacy/Meeting 0-1	**2.521 (1.464, 4.342)**	**2.712 (1.356, 5.425)**
Girls		
VitD adequacy/Meeting 2-3	1.000 (reference)	1.000 (reference)
VitD adequacy/Meeting 0-1	0.873 (0.247, 3.085)	0.495 (0.049, 5.012)
VitD inadequacy/Meeting 2-3	0.797 (0.377, 1.689)	0.994 (0.292, 3.388)
VitD inadequacy/Meeting 0-1	0.783 (0.357, 1.720)	0.813 (0.229, 2.889)
Age		
6~12 years		
VitD adequacy/Meeting 2-3	1.000 (reference)	1.000 (reference)
VitD adequacy/Meeting 0-1	1.163 (0.528, 2.558)	0.901 (0.233, 3.480)
VitD inadequacy/Meeting 2-3	1.282 (0.794, 2.069)	2.078 (0.983, 4.392)
VitD inadequacy/Meeting 0-1	**1.979 (1.197, 3.272)**	**3.234 (1.497, 6.985)**
13~18 years		
VitD adequacy/Meeting 2-3	1.000 (reference)	1.000 (reference)
VitD adequacy/Meeting 0-1	0.250 (0.027, 2.289)	0.254 (0.028, 2.329)
VitD inadequacy/Meeting 2-3	1.230 (0.462, 3.277)	1.137 (0.425, 3.044)
VitD inadequacy/Meeting 0-1	1.015 (0.384, 2.683)	0.892 (0.336, 2.372)

Model adjusted for age, sex, pubertal status, maternal education, paternal education, parental obesity, parental history of metabolic disorders, family income, tobacco exposure, and blood sampling season.

Abbreviations: CI, confidence interval; OR, odds ratio; MUO, metabolic unhealthy obesity; VitD, vitamin D.

Supplementary Table S4. Combined associations of serum vitamin D status and the number of guidelines met with general obesity and MUO after excluding the individuals that missing covariates values (n=4278).

	Crude	Model 1	Model 2
	OR (95%CI)	OR (95%CI)	OR (95%CI)
General obesity			
VitD adequacy/Meeting 2-3	1.000 (reference)	1.000 (reference)	1.000 (reference)
VitD adequacy/Meeting 0-1	1.152 (0.543, 2.442)	1.206 (0.567, 2.566)	1.125 (0.524, 2.412)
VitD inadequacy/Meeting 2-3	1.390 (0.880, 2.196)	1.586 (0.999, 2.517)	1.538 (0.961, 2.462)
VitD inadequacy/Meeting 0-1	1.576 (0.994, 2.499)	**2.114 (1.304, 3.429)**	**2.080 (1.276, 3.389)**
P-trend	0.031	0.001	0.001
MUO			
VitD adequacy/Meeting 2-3	1.000 (reference)	1.000 (reference)	1.000 (reference)
VitD adequacy/Meeting 0-1	0.910 (0.281, 2.950)	0.870 (0.267, 2.831)	0.812 (0.248, 2.656)
VitD inadequacy/Meeting 2-3	**2.136 (1.111, 4.106)**	**2.184 (1.130, 4.221)**	**2.178 (1.118, 4.241)**
VitD inadequacy/Meeting 0-1	**2.502 (1.298, 4.824)**	**2.472 (1.251, 4.883)**	**2.498 (1.261, 4.951)**
P-trend	0.001	0.003	0.002

Model 1 adjusted for age, sex;

Model 2 was further adjusted for pubertal status, maternal education, paternal education, parental obesity, parental history of metabolic disorders, family income, tobacco exposure, and blood sampling season.

Abbreviations: CI, confidence interval; OR, odds ratio; MUO, metabolic unhealthy obesity; VitD, vitamin D.

Supplementary Table S5. Sensitivity analyses of combined associations of serum vitamin D status and the number of guidelines met with general obesity and MUO.

	Model 1[a]	Model 2	Model 3
	OR (95%CI)	OR (95%CI)	OR (95%CI)
General obesity			
VitD adequacy/Meeting 2-3	1.000 (reference)	1.000 (reference)	1.000 (reference)
VitD adequacy/Meeting 0-1	0.925 (0.442, 1.934)	0.921 (0.440, 1.930)	0.910 (0.434, 1.907)
VitD inadequacy/Meeting 2-3	1.378 (0.897, 2.117)	1.322 (0.860, 2.032)	1.313 (0.854, 2.019)
VitD inadequacy/Meeting 0-1	**1.828 (1.169, 2.861)**	**1.729 (1.104, 2.708)**	**1.743 (1.113, 2.730)**
P-trend	0.001	0.003	0.002
MUO			
VitD adequacy/Meeting 2-3	1.000 (reference)	1.000 (reference)	1.000 (reference)
VitD adequacy/Meeting 0-1	0.643 (0.205, 2.020)	0.643 (0.204, 2.023)	0.628 (0.199, 1.981)
VitD inadequacy/Meeting 2-3	**1.843 (1.020, 3.330)**	1.758 (0.972, 3.179)	1.745 (0.964, 3.159)
VitD inadequacy/Meeting 0-1	**2.163 (1.177, 3.978)**	**2.029 (1.102, 3.735)**	**2.062 (1.119, 3.799)**
P-trend	0.002	0.006	0.004

[a] Model 1 based on the data after filled in missing covariates values by multiple imputation method.

Model 1 adjusted for age, sex, pubertal status, maternal education, paternal education, parental obesity, parental history of metabolic disorders, family income, tobacco exposure, and blood sampling season.

Model 2 was further adjusted for vitamin D supplements use.

Model 3 was further adjusted for dietary vitamin D intake (daily intake of milk, eggs, and meat).

Abbreviations: CI, confidence interval; OR, odds ratio; MUO, metabolic unhealthy obesity; VitD, vitamin D.

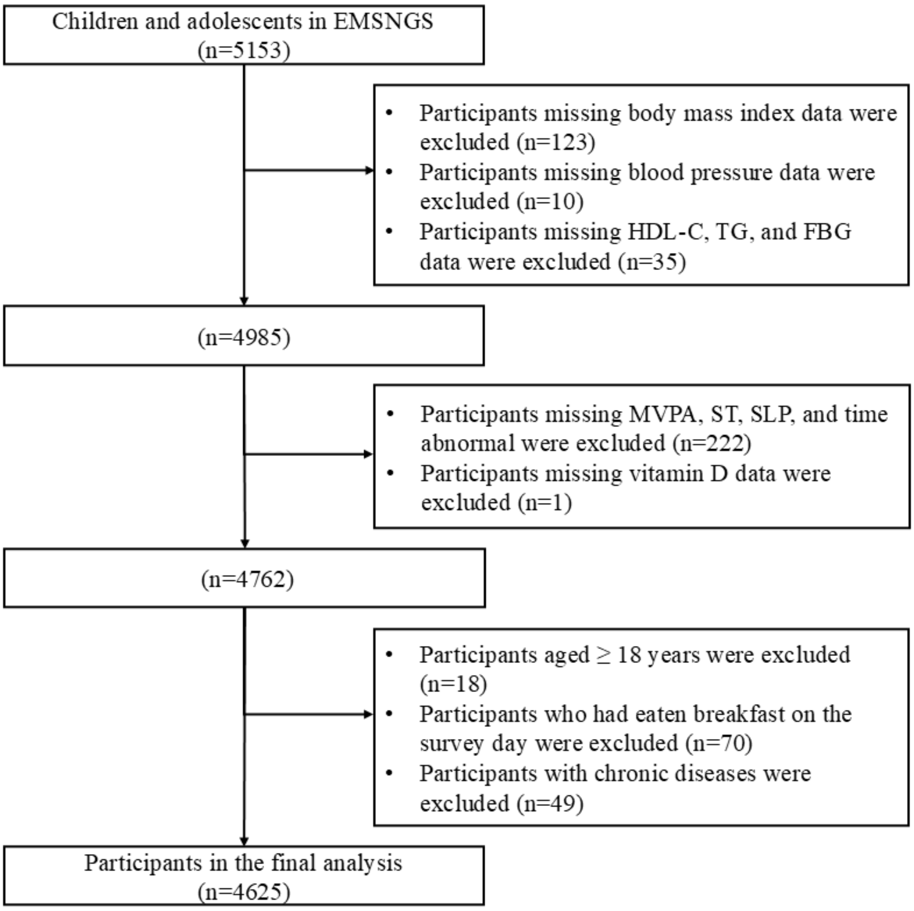

Supplementary Figure S1. Flowchart for the participants' selection.

Abbreviations: EMSNGS, Evaluation and Monitoring on School-based Nutrition and Growth in Shenzhen; FBG, fasting blood glucose; HDL-C, high-density lipoprotein cholesterol; MVPA, moderate-to-vigorous physical activity; ST, screen time; SLP, sleep duration; TG, triglyceride.

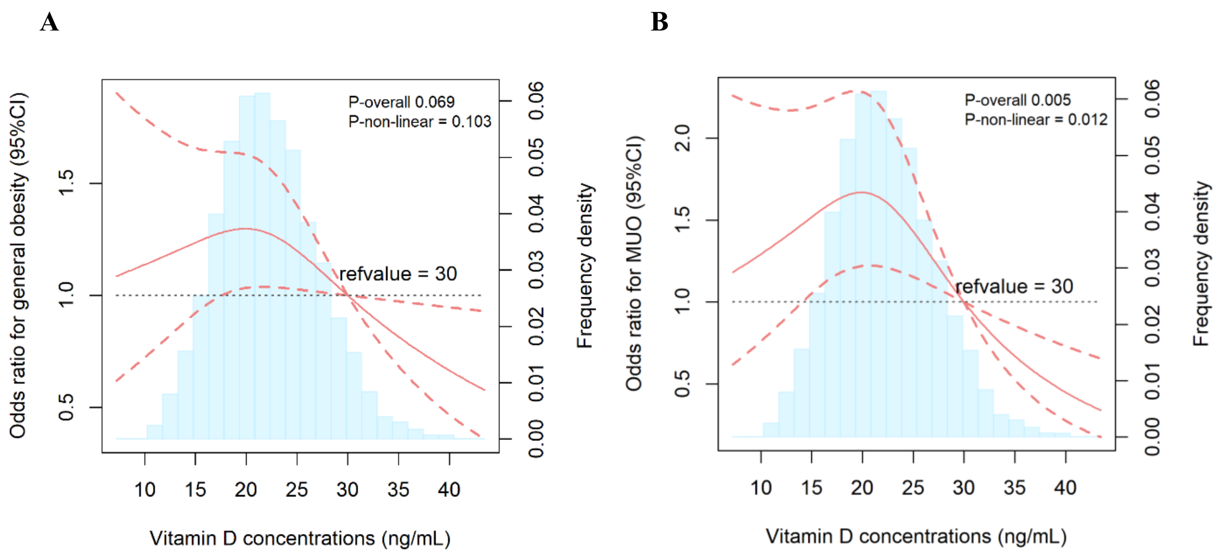

Supplementary Figure S2. Nonlinear dose-response relationship between vitamin D concentrations and general obesity or MUO in children and adolescents. (A) General obesity; (B) MUO.

Model adjusted for age, sex, pubertal status, maternal education, paternal education, parental obesity, parental history of metabolic disorders, family income, tobacco exposure, and blood sampling season.

Abbreviations: CI, confidence interval; MUO, metabolic unhealthy obesity.

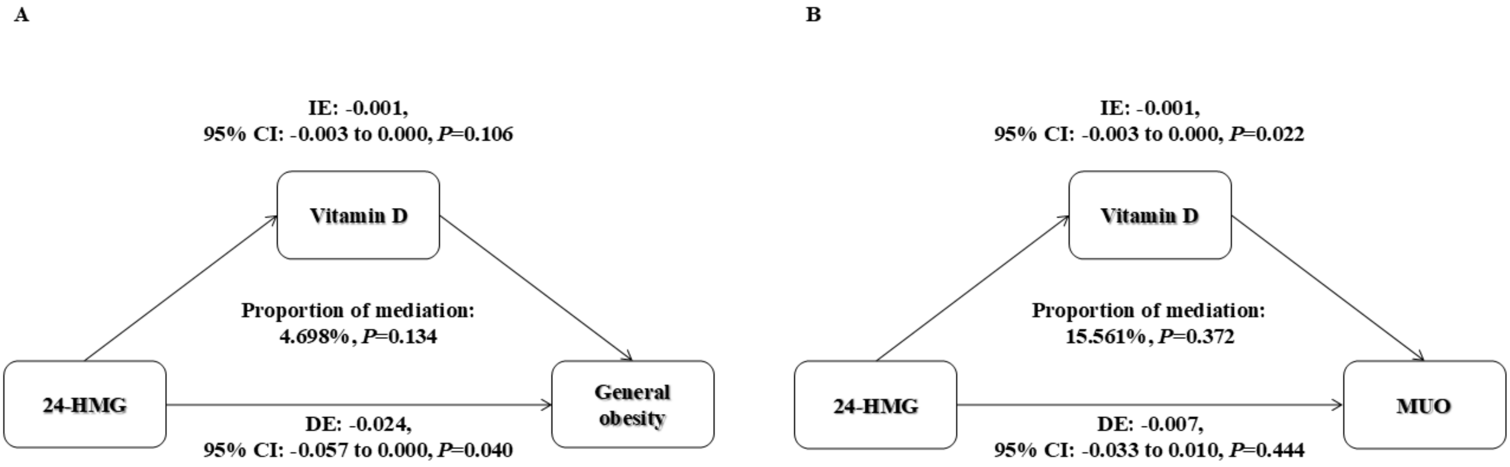

Figure S3. Parallel mediation analyses of the association between adherence to 24-HMG and general obesity or MUO mediated by vitamin D concentrations. (A) General obesity; (B) MUO.

Model adjusted for age, sex, pubertal status, maternal education, paternal education, parental obesity, parental history of metabolic disorders, family income, tobacco exposure, and blood sampling season.

Abbreviations: 24-HMG, 24-Hour Movement Guidelines; CI, confidence interval; DE, direct effect; IE, indirect effect; MUO, metabolic unhealthy obesity; Proportion of mediation = IE/DE+IE.
